# Promoting students’ safety and wellbeing: ethical practice in schools

**DOI:** 10.1007/s13384-022-00567-8

**Published:** 2022-08-30

**Authors:** Anne Graham, Antonia Canosa, Tess Boyle, Tim Moore, Nicola Taylor, Donnah Anderson, Sally Robinson

**Affiliations:** 1 Centre for Children and Young People, Southern Cross University, Gold Coast, QLD Australia; 2 Institute of Child Protection Studies, Australian Catholic University, Melbourne, VIC Australia; 3 Faculty of Law, Children’s Issues Centre, University of Otago, Dunedin, New Zealand; 4 School of Psychology, Charles Sturt University, Albury, NSW Australia; 5 College of Nursing and Health Sciences, Flinders University, Adelaide, SA Australia

**Keywords:** Schools, Students, Safety, Wellbeing, Ethics, Practices

## Abstract

Although ‘child safety’ is now a national policy priority in Australia, there is little research exploring the *practices* in schools that contribute to children and young people’s felt sense of safety and wellbeing. Drawing on a mixed-method Australian Research Council (ARC) Discovery project, this article presents findings from interviews with school staff (*N* = 10), leaders (*N* = 5) and nine focus groups with students (*N* = 58), in primary and secondary schools in three Australian states (New South Wales, Victoria and South Australia). We employ relational ethics, recognition theory and the theory of practice architectures to explore practices at school that support student wellbeing and safety. The findings contribute significantly to understanding the ‘bundled’ nature of current practices and the conditions that enable and constrain these. Close attention to these findings is critical as schools seek to operationalise the National Child Safe Principles and refine ongoing safeguarding procedures. The findings have informed the development of an online survey that is currently testing, on a much larger scale, which elements of ethical practice are most positively associated with students’ safety, wellbeing and recognition at school.

## Introduction

The increased focus in recent years on safety and wellbeing within schools in Australia, amplified by the Royal Commission into Institutional Responses to Child Sexual Abuse (hereafter ‘Royal Commission’), has resulted in numerous legislative and policy responses aimed at safeguarding children and developing child-safe organisations (Powell et al., 2020). The Royal Commission recommended all Australian institutions engaging in child-related work be required to implement the standards incorporated into the *National Principles for Child Safe Organisations 2018*. State and territory governments are required to report progress by the end of 2022. Consequently, schools and systems across every jurisdiction are working to ensure procedures and processes that embed child-safe cultures, and practices are consistent with the National standards and principles.

Current legislative and policy efforts are critically important. However, ongoing attention is needed to ensure they do not inadvertently reduce children’s safety to compliance-based imperatives that fail to create the cultural conditions necessary to promote their wellbeing (Powell et al., 2020). Historically, procedural documents providing child-safe guidance have foregrounded reporting and responding to disclosures, selection and recruitment, staff education, training and supervision, and risk management (Palmer et al., 2016). In the past, schools have reported that the administrative burdens associated with policy compliance around child protection and safeguarding resulted in teacher burn-out (Jeffrey et al., 2013). Yet research into children’s wellbeing in schools highlights the importance of positive relationships with self, teachers, friends, peers and significant others (Graham et al., 2017). Understanding the cultural conditions necessary for every student to thrive, feel like they belong, are safe and connected at school is a critical and ongoing challenge within education (Fogelgarn & Burns, 2020).

## What we know about safety and wellbeing in schools

Extant literature on safety in schools tends to focus on risk management of bullying and violence (McEachern et al., 2005; Thompson, 2019), online safety (Waller, 2017) and student–teacher relationships (Öhman, 2017). Child protection and abuse prevention discourses, along with growing anxiety around physical contact between adults and students, have given rise to a culture of fear for teachers (Taylor et al., 2016) and the development of ‘no touch’ policies that focus on teachers’ self-regulation (Öhman, 2017). Keeping children safe has been addressed by implementing safeguarding policies and procedures in this risk-sensitive environment. While mitigating risk is necessary, questions about whether such responses are enough are warranted.

Young people’s wellbeing and safety are connected to the relational context they live in and the relationships they establish at schools (Graham et al., 2018). This research demonstrates a connection between relationships and recognition characterised by mutual experiences of being cared about, respected and valued (Graham et al., 2017; Simmons et al., 2015). Though schools have increasing tasks connected to safeguarding procedures (Guidetti et al., 2018), acknowledgement of the critical role teachers play in fostering positive relationships with students and in creating the cultural conditions necessary for children and young people to feel happy, safe and connected at school is also increasing. Such findings resonate with research involving young people that indicates the relational context is closely linked with safety within child and youth-focussed organisations, including schools (Moore et al., 2018; Robinson & Graham, 2020). When asked how to manage risks and what they need to be safe and feel safe, children identified the importance of relationships with trusted adults or peers. Autonomy and control over their environments and opportunities to influence decisions that affect their lives were also perceived to increase their safety from harm (Moore et al., 2018).

## Theoretical framework

Ethical practice in this research is understood as the way ethical values are operationalised within human service settings. As such it is inextricably linked to the relational environment and to notions of professionalism (Thomas, 2012) and advocacy (Nastasi & Naser, 2014). The theoretical framework informing the study comprises three critical participatory elements, namely children’s rights (Wall, 2010), recognition theory (Honneth, 1995) and the theory of practice architectures, hereafter TPA (Kemmis et al., 2014).

The research situates ethical practice alongside children’s rights (Wall, 2010) by recognising children as persons worthy of dignity, status and voice (Canosa & Graham, 2020; Spyrou, 2018). Children and young people are understood as having agency and the right to both protection *and* participation in accordance with their evolving capacities (Taylor, 2006). This is critical given children’s understandings of ethics and ethical practice have been historically marginalised. Wall (2010) argues that ethical thinking is adult centric and that children are measured against adult norms within a context of citizenship that excludes them based on the notion of ‘dependency’. To view children as moral agents, Wall (2010) calls for a ‘profound ethical restructuring’ inclusive of children’s lived experiences, in which identity, diversity and difference are fundamentally recognised.

Recognition theory provides an essential lens for examining how human interactions contribute to identity formation—how one sees or relates to oneself—including self-confidence, self-respect and self-esteem (Honneth, 1995). Building on recent research with school students (Graham et al., 2017; Thomas et al., 2016), recognition is fundamental to individual and group identity via three dimensions: (a) being cared for; (b) being respected and (c) being valued. Turney (2012) argues that relationship-based practices, including recognition, respect and reciprocity, are at the heart of ethical engagement and moral decision making when working with children and young people. Utilising Honneth’s (1995) recognition theory enabled this inquiry to move beyond a descriptive analysis of relationship-based practices to a deeper understanding of whether and how these potentially mediate children’s safety and wellbeing.

Applying a neo-Aristotelian view of *praxis*, understood as “action that aims for the good of those involved and for the good of humankind” (Kemmis et al., 2014, p. 26), TPA sits comfortably alongside children’s rights and recognition theory, as well as relational ethics. TPA provides a site ontological perspective of practices by considering ways they are shaped (enabled and constrained) by conditions found at or brought to the site in which they unfold. From a TPA perspective, practices comprise words and ideas (sayings), actions and activities (doings), and relationships with self, others and the world (relatings) (Kemmis et al., 2014). The sayings, doings and relatings (practice elements) are bundled and co-occur within particular locations in physical space–time (Schatzki, 2002). As such, a practice cannot be reduced to any one of these elements alone (Kemmis & Grootenboer, 2008). TPA affords a theoretical and methodological resource for understanding education and professional practice—including challenging problematic practices and considering possibilities for transformative actions (Mahon et al., 2017).

In this research, we hear directly from students about ethical practices that contribute to their safety and wellbeing at school and bring their perspectives into dialogue with teachers and principals. Within the broader focus on children’s rights, the study has taken a distinctive approach in drawing together relational ethics, recognition theory and TPA to explore the ethical dimension of practices that support children’s fundamental right to feel safe and well and the conditions that enable and constrain these practices.

## The study

The present study has drawn from a large-scale mixed-method Australian Research Council (ARC) Discovery Project to strengthen knowledge, policy and practice concerning ‘child safe’ organisations by examining the role of ethical practice in improving children and young people’s safety and wellbeing. The research adds to increasing evidence linking young people’s safety and wellbeing with positive relationships (Anderson & Graham, 2015). It builds on the premise that schools are not just tasked with risk detection and mitigation but also working with, and empowering, students to proactively engage in practices that support their own safety and wellbeing, and also to seek help when needed (Smallbone, 2017). Unless children experience environments where emotional safety is paramount, they struggle to learn (Nowacka-Dobosz et al., 2019). Here, we report findings from the qualitative phase involving interviews with school staff (*N* = 10), leaders (*N* = 5) and nine focus groups with students (*N* = 58) in primary and secondary schools in three Australian states (New South Wales, Victoria and South Australia). This phase addressed the following research question: *How do children and practitioners in different institutional settings understand and experience ‘ethical’ practice with regard to children’s safety and wellbeing, and what do they perceive to be the enablers and barriers?*

## Methodology

To answer the research question, elements of the theoretical framework were employed as a methodological resource to reveal how practices, in this case, ethical practices, are experienced and understood in school sites. This site ontological approach provided a lens for *zooming in* on how these practices are experienced and for *zooming out* (Nicolini, 2012) to make sense of the practices by considering ways they are enabled and or constrained by conditions found at, or brought to, the site. This methodology critiques individualistic epistemic understandings of practices by acknowledging that people encounter each other in intersubjective (relational) spaces and that these spaces are already arranged in particular ways by conditions found at, or brought to, the site (Kemmis et al., 2014). In this research, hearing directly from students and staff about their lived experiences has facilitated insights into the ethical dimensions of practices, the ways these were enabled and constrained, and their implications for future actions. The research team also recruited and collaborated closely with a Young People’s Advisory Group (YPAG). The YPAG assisted with the phrasing and format of the focus group questions and the development of the mapping activities to guide the discussion during the focus groups.

 Ethics aspects of the research were approved was obtained from the lead University’s Human Research Ethics Committee (approval number: ECN-19-047) and relevant government and non-government school systems (approval numbers: 2019-759078; 2019-0611). Informed consent was obtained from students and staff, as well as from parents/carers. Core ethical principles were followed throughout the research process, informed by the International Ethical Research Involving Children (ERIC) Charter and Guidance (Graham et al., 2013).

### Selecting the sites

Selection of specific research sites was guided by (a) different states, each with particular jurisdictional policy imperatives; (b) different areas of geographical remoteness, as determined by the Australian Bureau of Statistics (Australian Bureau of Statistics, n.d.); and (c) willingness of the school to participate in the research. Maximum variation was sought in terms of school systems including one combined (including both primary and secondary grades) government school in an outer regional/remote area in South Australia (17 students and 3 staff); two Catholic schools in a regional area in New South Wales (20 students and 6 staff); and one combined Independent school in a major metropolitan area in Victoria (21 students and 6 staff). Diversity was sought in terms of school size, socioeconomic status, and geographic and cultural characteristics. Given ongoing restrictions, lockdowns and home schooling associated with the COVID-19 pandemic, recruitment of participants through schools was challenging. The interviews and focus groups in three of the schools were facilitated face to face and in one of the schools these were facilitated online via Zoom.

### Data collection: zooming in on the practices

Nine focus groups were conducted across four schools in three states with a total of 58 students. Student numbers ranged between 5 and 7 across each of the nine focus groups and included students of similar ages in the primary schools (Year 6 students, aged 11–12 years) and secondary schools (Year 7–9 students, aged 13–15 years and Year 10–12 students, aged 16–18 years). Two researchers co-facilitated the 60-min focus groups. After introductions and reiterating key issues of consent and confidentiality, each focus group commenced with an invitation to describe the ‘happenings’ at this school—what would they say to a friend who might be interested in enrolling? This activity preceded a mapping activity (using post-it notes) in which students identified practices that helped, or did not, in feeling happy, safe and well (mapping activity 1). The researchers invited students to reflect on practices they considered as having an ‘ethical’ dimension, elaborating where possible on how they understood the word ‘ethical’ (mapping activity 2). The students then suggested changes to practices that might support students to be happy, safe and well at their school (mapping activity 3).

Interviews (*N* = 15) were conducted at primary and secondary schools across the three geographical locations. One principal/deputy principal and two other staff members in each school were invited to participate in the interviews. The semi-structured interviews each took between 40 and 60 min, and participants were asked to describe the school they worked in, their role and level of experience. Following this, and similar to the student focus groups, staff were asked to reflect on the practices in their school that support the safety and wellbeing of students. Staff were then asked to identify and elaborate on the ethical dimensions of these practices.

### Data analysis: zooming out to make sense of the practices

**Fig. 1:**
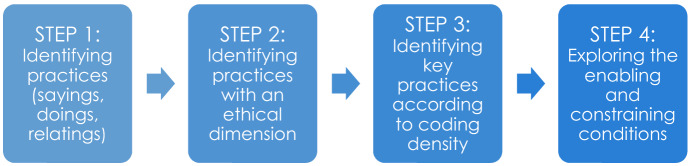
Data analysis stages

All interviews and focus groups were audio recorded with participants’ consent and subsequently transcribed, coded and analysed using QSR NVivo12, qualitative data management and analysis software (Bazeley & Jackson, 2013). The additional written data collected through the mapping activities were also transcribed or photographed and coded in NVivo. Initial themes (identified as tree nodes in NVivo) were developed from the questions that guided the interview and focus group schedules and were coded using the auto-code function in NVivo. This initial coding was followed by more in depth, manual coding performed in four steps (see Fig. 1):

1. Deductive coding was employed to identify data assigned to predefined themes or nodes according to TPA. The practice elements identified were grouped into words and ideas (sayings), actions and work (doings) and relationships and power (relatings).
2. Inductive coding was employed to identify practices identified by participants as having an ‘ethical’ dimension. In this case, students were asked to nominate practices they considered to be ‘the right thing to do’. This process required consideration of all the elements of our theoretical framework.
3. A coding density analysis was carried out in NVivo to identify practices mentioned most often by participants, discussed in the findings of this article. It is important to note that a coding density analysis in NVivo reports the number of times a theme is identified in the data and may include multiple mentions by a single participant.
4. Data were coded to identify the enabling and constraining conditions that facilitate or hinder ethical practice.

Data analysis involved constant comparison and reviewing of the text assigned to each theme/node. Visual representations such as mind maps were also helpful for organising and refining themes and seeking feedback from the YPAG members.

## Findings

Several practices were identified as contributing to children and young people’s felt sense of safety and wellbeing. These are summarised in Table 1 as words and ideas (sayings), actions and work (doings), relationships and power (relatings), as well as the enabling and constraining conditions that hold these practices in place.

**Table 1:** Exploring ethical practice through the theory of practice architectures

Practices	Conditions	
Sayings: words and ideas (cognitive)	Cultural discursive	
	Enabling	Constraining
Positive and encouraging language	Growth rather than deficit language in interactions with students Appropriate behaviour management strategies	Teacher bias, peer bullying and/or discrimination; teachers’ inappropriate use of authority in the classroom
Student voice and agency	Appropriate mechanisms in place to encourage student voice and participation in decision making (e.g. Student representative councils, Year Coordinators, classroom practices such as vent diaries; and student-driven initiatives)	Absence of student voice mechanisms; school culture stifles voice and agency
Reflexivity	Staff are supported to manage and critically reflect on ethical challenges (e.g. debriefing time, mentoring, leadership support) to support students’ safety and wellbeing at school	Lack of time to critically reflect; lack of support from leadership and/or other staff

Doings: actions and work (physical)	Material economic	
	Enabling	Constraining
Appropriate behaviour management strategies	Designated welfare/wellbeing officer and/or Year Coordinator; Student management systems (e.g. SchoolWorx); restorative practices; effective communication with parents; sit & think strategies	Inconsistency in behaviour management practices; lack of communication with students and families
Safe spaces for students	Consistent classroom routines; small classes with same teachers; year groups; libraries	Staff turnover; resourcing; compliance obligations
An authentic child-centred culture (child at the centre)	Leaders who promote a child-centred culture and empower staff to work in child-centred ways. Co-creation of values with students and staff to promote joint ownership. Recruitment, induction and professional development that contributes to child-centred cultures. Legal and compliance obligations that support student safety and wellbeing	Compliance and administrative obligations that prevent the development of a child-centred organisational culture; school values are not co-created with students and staff; lack of training and professional development for staff

Relatings: relationships and power (affective)	Social political	
	Enabling	Constraining
Positive relationships with students	Staff are supported to build positive relationships with students based on mutual trust and respect. Teachers demonstrate personal qualities suited to working effectively with young people. Leaders model positive relationships with students	Lack of time, feeling overburdened; staff turnover; teachers not suited to working with children
Care and respect for students	Staff convey interest and respect towards young people, for who they are as human beings and their associated rights	Lack of time, feeling overburdened; staff turnover; teachers not suited to working with children
Equality, fairness and inclusiveness	Staff are encouraged to treat students equally, fairly and inclusively	Teacher bias; peer bullying and/or discrimination; teachers’ inappropriate use of authority in the classroom

**Fig. 2:**
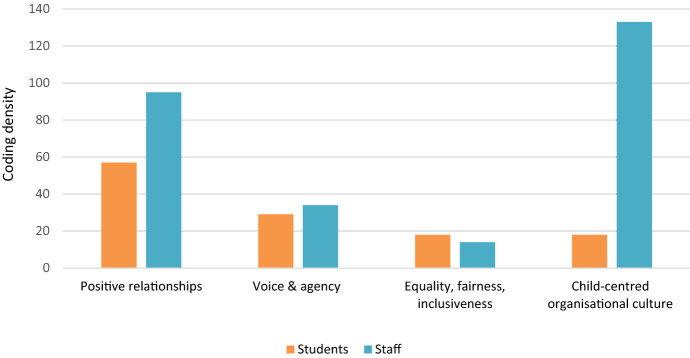
Coding density by participant type in schools. *The ‘staff’ data include principals and deputy principals

Participants identified several practices as having an ethical dimension when viewed through a lens of ‘what is the most ‘right’ thing to do?’ (Step 2). A coding density analysis of these practices in NVivo (Step 3: Fig. 1) revealed four categories of practices with an ethical dimension. These were: (i) building positive relationships with students; (ii) promoting student voice and agency; (iii) being equal, fair and inclusive with students; and (iv) contributing to an authentic child-centred organisational culture. Given our interest in student voice, the findings of this analysis are presented according to the coding density of the student data (see Fig. 2).

Although we acknowledge practices and conditions are invariably ‘bundled’ (Schatzki, 2002), these are discussed here in the findings. The concomitant conditions that enabled and constrained these practices are then presented in the discussion section and synthesised in Table 1.

### Building positive relationships with students

‘Positive relationships’ were the most prominent theme in the student data (see Fig. 2). Students described these relationships primarily in recognition terms, aligned with being cared for, respected and valued. Central to these relationships were trust issues, with students perceiving that staff who were encouraging and took the time to build relationships with students could be trusted. One younger participant described such trust in terms of feeling comfortable and safe talking to a teacher she knows well: “I feel that if I were to go to a teacher, I would go to a teacher that I’ve had in my classroom, that I know is encouraging and will be there to help me through any hard time” (Girl, 11–12 years, Primary School). Several students expressed views about how relationships between students and teachers, based on mutual trust and respect, were more likely to result in shared authority in the classroom: “Young teachers are more creative, they let you have more freedom, use your voice and how can you change things like that” (Girl, 16–18 years, Secondary school A1). Some students also implied that staff who are ‘relatable’ and have an aptitude for working with children and young people are more likely to instil trust and act in ways that positively influence their wellbeing and safety: “Some teachers are easier to talk to than others because other teachers are really strict and if you say something, then they probably just disagree” (Girl, 13–15 years, Secondary school A1). Relationships with peers were also perceived as necessary for feeling safe and well at school. Valuing, supporting and encouraging each other were viewed as an integral aspect of ‘doing what is right’ for others’ safety and wellbeing, as this younger student in Year 6 explains: “Friends are always there for you when you need them, having a good friend group to be around that support you” (Boy, 11–12 years, Primary school A).

For staff, too, relationships featured strongly in practices that have an ethical dimension (being the second most frequently mentioned after organisational culture—see Fig. 2). They described solid and positive relationships with students characterised by rapport, trust and respect as central: “Relationships are key to everything. They’re the bottom line. If you don’t build those relationships, if you don’t have that trust, you, me, children, nothing else will happen” (Leader, Secondary school B1). Often, the practices associated with building trust were described in terms of ‘getting to know’ children, that is, understanding and valuing every students’ strengths, limitations, interests and capabilities: “But particularly for me in the classroom to know that I value them for who they are as an individual. I’m interested in them and what they do on weekends, them as an individual, not just about academics” (Teacher, Primary school A).

Both staff and students underlined that having leaders who model positive relationships was important. Some students felt that when leaders failed to build positive relationships with students, this harmed their safety and wellbeing:

…it would be a lot more helpful if we had a Principal that had relationships with students, instead of sitting up in the high office, you hardly see him and the only time you see him is if you’re in trouble or at assembly. I feel like you’d be a lot more happy and safe if you were greeted each morning. (Boy, 15-18 years, Secondary school A1)

### Promoting student voice and agency

As evidenced in the coding density illustrated in Fig. 2, students perceived that being heard and being able to contribute to and influence decisions were also important in supporting their wellbeing and safety. Students identified several existing practices designed to elicit student voice, including surveys, Student Representative Councils and Year Coordinators. They highlighted, however that practices in classrooms that enabled them to have a voice and influence decisions were significant (see Table 1). In providing opportunities for student voice, teachers demonstrated they valued students’ opinions, as the following quote exemplifies:

Yesterday I was given an assignment from one of my teachers, and one of the questions was if the teacher was going to change their ways of teaching, what would be your preference on what would they do? How will they do it? I told him that he thought it was good feedback, so they might take it into account for next year. (Boy 1, 13-15 years, Secondary school A1)

Students identified the culture within schools as enabling and or constraining their voice and agency (see Table 1). Students felt that having a say and influencing how things are done in school contributed to a felt sense of ownership. For example, in one primary school, students underlined the importance of having the opportunity to regularly put forward ideas and work towards shared explicit goals (which might range from reducing lunch box rubbish to community outreach initiatives). Conversely, older students often discussed how prevailing conditions or arrangements in their school can sometimes constrain their voice and agency: “We’ve put forward ideas, but then like the teacher never really does anything about it” (Girl 2, 16–18 years, Secondary school A1).

Practices linked to ‘voice and agency’ were not discussed as often by staff (see Fig. 2). When these *were* raised, staff mostly referred to how students are encouraged to voice their opinions. They rarely considered students’ ability or right to some autonomy or to act in their interests. Teachers primarily talked about facilitating student voice, but without reference to any need to act on this: “The student voice is very important to us, and we offer different opportunities for everyone to have a say in what’s happening in their learning” (Leader, Secondary school C1). On the other hand, several staff indicated that valuing students’ opinions is a dimension of ethical practice which contributes to safety and wellbeing:

I think that hearing what a student has to say is part of ethical practice—a lot of time they just want to talk and feel like they are being heard—sometimes by the end of this they feel better and don’t even need you to ‘do’ anything. (Teacher, Secondary school C)

This evidence suggests that how voice and agency were understood and conceptualised across students and staff differed considerably. While staff mainly discussed ways to promote student voice, young people highlighted the importance of acting on their perspectives and being invited to influence decision making.

### Being equal, fair and inclusive with students

Students flagged issues related to being treated equally, fairly and inclusively by teachers and peers as necessary in feeling happy, safe and well at school. This practice area was mentioned more frequently in the student than the staff data (see Fig. 2). Creating a welcoming, inclusive environment was viewed as foundational and the attitude of teachers integral in enabling the cultural conditions for this practice: “Having a caring attitude, making sure that everyone feels welcomed and safe in the school environment and kids learning to be kind to everybody” (Girl 1, 11–12 years, Primary school A). Conversely, bias and discrimination—enacted by peers and/or teachers—was identified as a constraint in achieving the fairness and inclusivity necessary for a felt sense of safety and wellbeing. Examples of such bias and discrimination perceived by students included peer bullying and/or discrimination and teachers’ inappropriate use of authority in the classroom (see Table 1). In Mapping activity 2, where students elaborated on practices they perceived to have an ethical dimension, issues of recognition (notably being cared for and respected), featured strongly in enabling the conditions for equality, fairness and inclusivity: “Respected, included, cared for, loved by everyone; teachers care for everybody, no one is left out;… everyone is welcome and friends with each other and collaborates together; everybody needs to be included no matter how you look/are” (Mapping activity 2, 11–12 years, focus group Primary school A).

While less prominent in the data, staff also referred to the ethical aspects of students being treated equally, fairly and inclusively. Central to enabling an ethical approach was clear, open and transparent communication with students and respect for their dignity. Conversely, staff perceived that *not* treating students with fairness and dignity was *un*ethical. As one Principal in a secondary school argued, students feel as though they are treated equally, fairly and inclusively when teachers genuinely value them and do what ‘is right’ consistently:

It’s about what’s right, and it’s not always popular, but I think if people hear that you are always doing what’s right, they then know that they can trust you, because when people tell you something, they want you to do what’s right. They’re scared, but they want you to do what’s right. That’s why they’re telling you because they know it’s wrong. (Leader, Secondary school B1)

At this stage of the analysis, it is evident that the practices identified and discussed above are inextricably linked or bundled. Without positive relationships, student voice and agency, equality, fairness and inclusivity, the possibilities for establishing a child-centred organisational culture are significantly diminished.

### Contributing to an authentic child-centred organisational culture

For this analysis, organisational cultures relate to the shared values, expectations and actions that are reinforced by both leadership/staff and students to shape how individuals and teams interact and relate to each other. While students did not refer explicitly to organisational culture, they often referred to the kind of school environment where they felt happy, safe and well. Such an environment was invariably tied to the importance they placed on relationships with staff and leaders, characterised by mutual ‘trust and respect’, ‘voice and agency’, ‘fairness, equality and inclusivity’.

For staff, organisational culture was the most prominent area of practice (see Fig. 2). The fostering of organisational cultures that promote students’ safety and wellbeing encompassed several practices which collectively contribute to an authentically child-centred culture—where consideration is given to the standpoint of the child, including what they experience, may need and know, and where staff demonstrate the centrality of this in everyday routine practice. Staff underlined the importance of a child-centred culture being led ‘from the top’ with many suggesting that practices around developing such a culture need to be understood and shared across the school, evidenced in the ethos or philosophy (the ‘way we do things around here’) and promoted by leaders who challenge and empower staff to work in a child-centred way: “I do think it comes down to that underlying culture of the school and the leadership of the school and what’s important to them and how all staff portray that*”* (Teacher, Primary school B). Similarly, school leaders pointed out that such a culture has to be supported by processes such as recruitment, induction, supervision and professional development that contribute to the same shared ethos and vision: “I just think if teachers have a common, known, clear vision of what we’re trying to do, then it works. When you’ve got people operating in silos or going rogue if you like, that’s when it falls down” (Leader, Secondary school B1). Recruitment practices, particularly, emerged as quite central in building and sustaining child-centred organisational cultures. These were inextricably bundled to practices around ‘relationships’ in that personal qualities of staff and their ability to work effectively with children and young people were viewed by many staff as central to *ethical* practice. School leaders, in particular, commented that working effectively with children and young people requires staff who are approachable, relatable and passionate about their job. Often leaders in schools discussed the difficulties in recruiting new teachers without having prior knowledge of their aptitude and disposition for working with children:

You want to build a team of like-minded people. We’re not employing people who don’t believe in what we believe in. … A lot of applicants will have all the credentials, but if they don’t have the soft skills, it’s not going to work. We want team players. We want people who love teaching because if you don’t love it, all the other pressures are going to grind you down. … The recruitment questions are around what we want and, if you don’t fit that mould, well, it’s not going to work. (Leader, Primary school B)

In addition, some staff across both primary and secondary schools emphasised that having leaders and colleagues who support them to reflect critically on complex issues was essential in effectively managing ethical challenges:

I mean when you make decisions around care and welfare of students, sometimes you can always ask yourself, are you doing the right thing? I always like to have a conversation with somebody else, particularly if it’s a tricky case or if there’s implications. I’ve got people in roles here that have more knowledge and experience than I do. So, I think just being able to bounce ideas and have that discussion is really important. I don’t think I would be able to go in and make a decision myself in those complex situations that you’re talking about. (Teacher, Secondary school A1)

Such findings suggest that building a child-centred organisational culture incorporates practices that may not always be articulated in ‘tick box’ review processes but rather are linked to the relational context of students’ learning environment. Step 4 of the analysis (Fig. 1) revealed enabling and constraining conditions shaping practices with an ethical dimension (Table 1). Following TPA (Kemmis et al., 2014), changing practices is contingent on the transformation of existing arrangements (conditions) that shape and hold them in place.

## Discussion and implications

The findings around key areas of practice identified above shed important light on the nuanced ways in which students and staff perceive what supports and constrains student safety and wellbeing at school. TPA provided a lens for ‘zooming in’ on their lived experiences of practices in schools and capturing student and staff perspectives on the ethical dimensions of these. In trying to understand how these practices are enacted, we explored how the cultural-discursive (sayings), material-economic (doings) and social-political (relatings) conditions enable and or constrain ethical practice at schools (Kemmis et al., 2014). In doing so, we were simultaneously ‘zooming out’ to make sense of the conditions that impact children’s safety and wellbeing (Nicolini, 2012). Table 1 shows how each practice is enabled and or constrained by certain context-specific conditions but have similarities across the schools in our sample.

The cultural-discursive conditions (‘sayings’) identified by students and staff as contributing to student wellbeing and safety were enabled by certain cultural-discursive conditions in the schools that privileged students’ voices; promoted positive language (moving away from deficit views of children); created opportunities to involve students in decision making (e.g. student representative councils, student-driven initiatives etc.); and supported staff to critically reflect on ethical challenges (e.g. debriefing time, mentoring and leadership support). Conversely, conditions identified as constraining ethical practices included: teacher bias and or discrimination; inappropriate use of authority in the classroom; absence of student voice mechanisms; and lack of time and support for staff to reflect critically on ethically challenging situations (see Table 1). Students thought that if adults took the time to build meaningful relationships with students, they were more likely to use their authority in the classroom appropriately.

The material-economic conditions (‘doings’) that enable ethical practices were discussed mainly by staff concerning the physical elements that contribute to child-centred school cultures. Adult participants discussed how certain practices enhanced the safety and wellbeing of students through, for example, a designated welfare/wellbeing officer and or Year Coordinator; a student management system to record student wellbeing concerns (e.g. SchoolWorx); restorative practices and classroom strategies (e.g. sit & think); and good communication with parents. Schools with leaders who promoted a child-centred culture and empowered staff to work in child-centred ways were more likely to use several of these practices to enhance student wellbeing and safety. Conversely, school compliance and administrative obligations were regarded as necessary but often took time away from developing meaningful relationships with students. These often-included systemic issues connected to curriculum requirements, assessment and reporting obligations (Table 1). Often staff felt ‘overworked’ and ‘overburdened’ and unable to nurture those positive relationships which have been identified as being so important to student wellbeing (see also Fogelgarn & Burns, 2020). Jeffrey et al. (2013) argue that increased academic and administrative demands on schools and teachers have posed challenges in maintaining the personal teacher–student relationships that form the basis for learning. Guidetti et al. (2018) argue that teacher burn-out results in deficiencies in the relational experience which may impact not only on the student–teacher relationship but also on the quality of student–peer interactions.

The social-political conditions (‘relatings’) were evidenced in the emphasis placed on meaningful relationships as one of the most critical dimensions of ethical practice leading to students’ felt sense of safety and wellbeing (see Fig. 2). The ‘relatings’ that young people discussed (particularly linked to being valued, respected, cared for and treated equally) were identified as essential in feeling happy, safe and well. The social-political conditions in schools that enabled such practices included the support (i.e. time and resources) received by staff to build positive relationships with students based on mutual trust, respect and equality, as well as teachers’ demonstrated personal qualities for working effectively and ethically with young people (see Table 1). Growing evidence suggests that students’ wellbeing and safety are closely linked to the positive relationships developed with teachers, friends, peers and significant others in schools (Graham et al., 2017, 2018; Powell et al., 2018). While similar themes emerged from the staff and student data, there are several interesting differences. Students identified positive relationships as the main facilitator of ethical practice. In contrast, staff and leaders placed a greater priority on child-centred organisational cultures and leadership as an essential condition to enable ethical practice (see Fig. 2). Staff and leaders also argued that building a school-wide shared understanding of positioning the child at the centre of their work was critical in achieving ethical practice. This was supported by processes such as recruitment, induction and professional development that equipped teachers to approach their work in child-centred and ethical ways (see also Tirri & Husu, 2002). Leaders stressed the importance of well-considered recruitment processes that explicitly identify personal qualities suited to working ethically with children and young people, underlining the role these play in a school culture where children and young people feel safe, happy and well. Regular training and staff professional development was also a necessary condition to enable ethical practices in schools (see Table 1). These findings are consistent with previous research that points to the importance of a more targeted approach to professional development in schools to equip teachers with the necessary tools to successfully negotiate ethically challenging situations (Tirri & Husu, 2002).

Students also emphasised the absence of trusting relationships as a constraining condition in achieving a felt sense of safety and wellbeing. Closely linked to this theme was the need to be treated equally, with fairness and dignity, which led to staff and students arguing that certain biased or discriminatory practices enacted by peers and or teachers were unethical and impeded students’ safety, happiness and wellbeing. Interestingly, students in this research (rather than staff) identified a lack of voice and autonomy as a barrier to feeling safe, happy and well (see Table 1). Students felt organisational cultures and rules within schools that limited their autonomy and placed undue pressure on them to perform, negatively impacted their wellbeing.

Considering this study’s broader theoretical interests in children’s rights, relational ethics and recognition theory, TPA was a useful analytical tool that unveiled the highly bundled nature of practices in schools that contribute to students’ safety and wellbeing. We have discussed the ethical dimensions (zooming in) of key areas of practice while also identifying the enabling and constraining conditions within the cultural-discursive (sayings), material-economic (doings) and social-political (relatings) realms that shape the professional practices in schools (zooming out). This process makes evident that if changes to practices are to be effected, then changes to the conditions must also be effected (Boyle & Wilkinson, 2018). Otherwise, short-term reactionary change (albeit well intentioned) is diluted by immutable conditions shaping contrary practices—in other words, ‘the way we do things around here’. In this regard, we suggest closer attention needs to be given to the perspectives offered by students concerning the ethical dimension of practices that support their wellbeing and safety and bring their perspectives into dialogue with those of adult participants. As Wall (2010) argues, we need to reimagine ethics through the experiences and perspectives of children yet given the historical adultcentrism prevalent in both educational policy and practice, we may still have a way to go in changing these conditions and transforming practices.

## Conclusion

This article discussed findings from interviews and focus groups with students and staff in primary and secondary schools across three educational systems, namely the government school system (SA), the Catholic school system (NSW) and the Independent school system (Vic). From a child rights-based perspective, it was necessary to hear directly from students in this research about ethical practices in building safety and wellbeing at school and bring their perspectives into dialogue with adult participants. The findings presented in this paper contribute significantly to understandings of current practices and the conditions that enable and constrain these. Close attention to these findings is particularly critical as schools currently seek to operationalise the National Child Safe Principles including refining ongoing safeguarding procedures. For these landmark policy developments to give rise to changed practices that improve children’s and young people’s safety and wellbeing, we argue that there is a pressing need to closely examine the conditions that may constrain, as well as enable, such changes. The findings reported here are from Phase 2 of a more extensive study. Phase 3 of this study is now examining, on a significantly larger scale, which of the identified practices are most positively associated with wellbeing and safety and the role that recognition (being cared for, respected and valued) plays in mediating these experiences.
